# Interaction of Factors Determining Critical Power

**DOI:** 10.1007/s40279-022-01805-w

**Published:** 2023-01-09

**Authors:** Richie P. Goulding, Simon Marwood

**Affiliations:** 1 Laboratory for Myology, Faculty of Behavioural and Movement Sciences, Vrije Universiteit Amsterdam, Amsterdam Movement Sciences, O|2 Labgebouw, Vrije Universiteit, De Boelelaan 1108, 1081 HZ Amsterdam, The Netherlands; 2 School of Health Sciences, Liverpool Hope University, Liverpool, UK

## Abstract

The physiological determinants of high-intensity exercise tolerance are important for both elite human performance and morbidity, mortality and disease in clinical settings. The asymptote of the hyperbolic relation between external power and time to task failure, critical power, represents the threshold intensity above which systemic and intramuscular metabolic homeostasis can no longer be maintained. After ~ 60 years of research into the phenomenon of critical power, a clear understanding of its physiological determinants has emerged. The purpose of the present review is to critically examine this contemporary evidence in order to explain the physiological underpinnings of critical power. Evidence demonstrating that alterations in convective and diffusive oxygen delivery can impact upon critical power is first addressed. Subsequently, evidence is considered that shows that rates of muscle oxygen utilisation, inferred via the kinetics of pulmonary oxygen consumption, can influence critical power. The data reveal a clear picture that alterations in the rates of flux along every step of the oxygen transport and utilisation pathways influence critical power. It is also clear that critical power is influenced by motor unit recruitment patterns. On this basis, it is proposed that convective and diffusive oxygen delivery act in concert with muscle oxygen utilisation rates to determine the intracellular metabolic milieu and state of fatigue within the myocytes. This interacts with exercising muscle mass and motor unit recruitment patterns to ultimately determine critical power.

**Supplementary Information:**

The online version contains supplementary material available at 10.1007/s40279-022-01805-w.

## Key Points

**Table:** 

Critical power represents the threshold intensity above which steady-state metabolism is no longer attainable, and within the last ~ 15 years, experimental data have emerged that illuminate its underpinning physiological determinants.
Here, we summarise these experimental data to demonstrate that critical power is a parameter of aerobic function that is affected by alterations in the capacities of each step in the oxygen transport and utilisation pathways.
Convective/diffusive oxygen delivery and intracellular oxygen utilisation rates interact with muscle fibre composition and motor unit recruitment profiles to determine the upper limit for steady-state exercise.

## Introduction

The determinants of exercise tolerance are of clear interest because of the strong relationships between exercise capacity and athletic performance [1, 2], health in the general population, and clinical outcomes in disease populations [3, 4]. Exercise intensity is, of course, a key factor that determines the tolerability of a given task. Moreover, for individuals or groups of individuals, partitioning the exercise intensity spectrum into domains where the physiological responses to a given task share common qualitative characteristics is an effective approach that can yield insight into the physiological determinants of exercise tolerance. Accordingly, the mechanisms of fatigue and determinants of exercise intolerance are not ubiquitous across the spectrum of exercise intensities [5]. However, above a particular individual-specific power output, the consistent feature of exercise intolerance (and hence, impending task failure) is the inability for pulmonary oxygen uptake ($\dot{V}$O_2_) and [lactate] (L^−^) to attain a steady state [6–9]. Thus, for each individual, there exists a range of intensities for which a steady state in pulmonary $\dot{V}$O_2_ is attainable, and a range for which it is not [6, 9–12], with the duration of sustainable exercise in the latter being significantly limited compared with the former. The threshold intensity that separates these two ranges of system behaviour, and its position relative to other landmarks of aerobic function (i.e. maximal $\dot{V}$O_2_ [$\dot{V}$O_2max_] and the lactate threshold), is therefore a fundamental determinant of the ability to sustain exercise [6, 13–15].

**Fig. 1:**
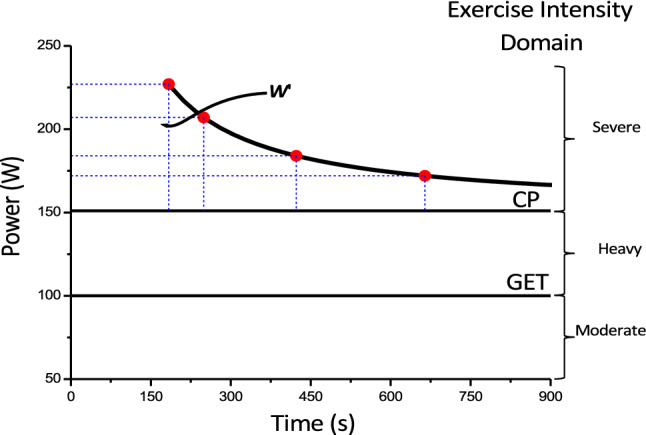
Hyperbolic power-duration curve that defines the sustainable duration of exercise in the severe-intensity domain. This hyperbolic relationship is defined by two parameters: the power asymptote, known as the critical power (CP), and the curvature constant *W*′ (denoted by the rectangular dashed blue lines above CP and expressed in kilojoules). Critical power defines the boundary between the heavy- and severe-intensity exercise domains and represents the highest power output for which a metabolic steady state may be attained. The W' comprises a fixed and finite volume of work that is expendable above CP. During severe-intensity exercise, task failure occurs when *W*′ = 0. *GET* gas exchange threshold

This threshold intensity can be determined by undertaking three to five high-intensity, constant-power output cycle ergometer tests to the point of task failure on separate days. The tests should be selected to last no less than 2 and no more than 15 min in duration [16–19], with the precise time to task failure and power output at which each test is conducted recorded. These durations are recommended for a valid determination of this intensity, as it is essential that $\dot{V}$O_2max_ is attained at the end of trial in order to meet the requirement for all prediction trials to be performed within the severe-intensity domain. When time to task failure is plotted against power output, the relationship is curvilinear, with the ability to sustain exercise falling away more rapidly at higher power outputs (Fig. 1). This power-time relationship is well described by a hyperbolic function [20], with an asymptote known as critical power (CP) and the curvature constant termed *W*' (i.e. W prime). This relationship is described by the following equation:

$$T=\frac{{W}^{{\prime}}}{P-CP},$$

where *T* is the tolerable duration and *P* is the power output of a given exercise task [6, 20, 21]. When intensity is measured in units of speed, the asymptote is termed critical speed (CS) and the curvature constant *D*’ (i.e. with units of distance). This power-time relationship appears to be a universal feature of high-intensity exercise tolerance, being apparent in every species [22–26] and mode of exercise (with appropriate units of force, torque or velocity [15, 27–30]) in which it has been studied. This relationship can also be converted to its linear equivalents, either with work plotted against time:

$$W=CP\cdot T+{W}^{^{\prime}},$$

where *W* is work, *CP* is the slope and *W*′ is the intercept of the equation, or with power plotted against the inverse of time:

$$P={W}^{^{\prime}}\cdot \left(\frac{1}{T}\right)+CP,$$

or

$$P=\frac{{W}^{^{\prime}}}{T}+CP,$$

where *CP* is the intercept and *W'* is the slope of the equation.

Since the seminal work by Prof. David Poole and colleagues in the late 1980s, it has been repeatedly demonstrated that CP reflects the upper limit at which a metabolic steady state can be sustained. The basis for this has been the ubiquity of steady-state behaviour of metabolic variables associated with aerobic function below, but not above, CP. For example, $\dot{V}$O_2_ rises to $\dot{V}$O_2max_ during exercise above, but not at or below, CP [6], accompanied by similarly inexorable trajectories of blood [lactate], [HCO_3_^−^] and pH [6, 31]. Such findings were subsequently confirmed in different populations, including the elderly [32], patients with chronic heart failure [33] and patients with chronic obstructive pulmonary disease (COPD) [34, 35], and healthy children [36]. More recently, non-invasive (^31^P-magnetic resonance spectroscopy, near-infrared spectroscopy) and invasive (i.e. muscle biopsy) studies have demonstrated the achievement of a steady state in the exercising muscle below, but not above, CP in muscle $\dot{V}$O_2_, [phosphocreatine] ([PCr]), [inorganic phosphate] [Pi], pH and muscle [lactate] [15, 31, 37]; for a review, see [8, 11, 12]. Critical speed (an analogue of CP) has also been demonstrated to be a critical threshold for motor unit recruitment patterns, with Copp et al. demonstrating that exercise above CS was accompanied by disproportionate increases in blood flow to type IIb/d/x fibres in the rat hind limb muscle [25].

Despite CP, and its analogues of external expression (i.e. critical speed, torque, force) being widely recognised as reflecting the threshold intensity above which a metabolic steady state cannot be sustained, its physiological antecedents have previously been obscure. Tables 1 and 2 detail interventional and observational approaches to understanding CP. Prior to the year 2010, intervention studies on CP were scant, and primarily confined to the effect of exercise training alongside additional measures of $\dot{V}$O_2max_ and the gas exchange threshold/lactate threshold only, although one of the earliest studies on CP did show an independent effect of O_2_ availability on CP (albeit in just two participants [21]). Nevertheless, such findings supported the notion of CP as a parameter of aerobic function [20]. In contrast, since 2010, multiple experimental approaches have revealed those factors that, directly or indirectly, determine CP. The purpose of the present review is therefore to examine the physiological and biochemical underpinnings of this fundamental parameter of exercise tolerance. Particular attention will be paid to evidence generated over the last 10–12 years demonstrating that CP is a key parameter of aerobic function that can be affected by any step in the O_2_ transport and utilisation pathway.

**Table 1:** Summary of studies that have altered critical power (CP) via chronic or acute interventions

Study	Population	Mode	Intervention	Effect on CP	Physiological effects of intervention	CP determination method
Moritani et al. [21]	H (2)	Upright cycling	Hypoxia (FiO_2_ = 0.09)	↓ CP (106 vs 214 W)		4CWR
Gaesser and Wilson [158]	ETM (2) HM (3)	Upright cycling	Endurance training (6 weeks)	↑ CP (228 vs 201 W)	↔ $\dot{V}$O_2peak_	4CWR
Gaesser and Wilson [158]	ETM (3) HM (3)	Upright cycling	HIIT (6 weeks)	↑ CP (254 vs 220 W)	↑$\dot{V}$O_2peak_	4CWR
Poole et al. [159]	HM (8)	Upright cycling	HIIT (7 weeks)	↑ CP (288 vs 325 W)	↑$\dot{V}$O_2peak_, ↑LT	5CWR
Jenkins and Quigley [160]	HM (12)	Upright cycling	Endurance training (8 weeks)	↑ CP (255 vs 196 W)	↑$\dot{V}$O_2peak_	3CWR
Hill [161]	HM (13) HF (11)	Upright cycling	Cadence (100 rpm vs 60 rpm)	↓ CP (195 vs 207 W)		4CWR
Serres et al. [162]	COPD (8)	Upright single leg knee extension	Endurance training (3 weeks)	↑ CP (1.8 vs 1.3 kg.s^−1^)	↑$\dot{V}$O_2peak_, ↑MVC	3CWR
Puente-Maestu et al. [38]	COPDM (27)	Upright cycling	Endurance training (6 weeks)	↑ CP (65 vs 58 W)	↑$\dot{V}$O_2peak_, ↓peak blood [La], ↓$\dot{V}$e_peak_	3CWR
Barker et al. [163]*	ETM (5) ATM (6)	Upright cycling	Cadence (100 rpm vs 60 rpm)	↓ CP (189 vs 297 W)		4CWR
Vanhatalo et al. [164]	HM (8) HF (1)	Upright cycling	HIIT (4 weeks)	↑ CP (255 vs 230 W)	↑$\dot{V}$O_2peak_, ↑GET	3MT
Miura et al. [165]	HM (6) HF (2)	Upright cycling	Heavy priming exercise	↑ CP (177 vs 169 W)		4CWR
Vanhatalo et al. [37]	HM (7)	Prone knee extension	Hyperoxia (FiO_2_ = 0.7)	↑ CP (18 vs 16 W)	↓Rate of change: muscle [ADP], [PCr], [Pi], pH; ↑*τ*_PCr_, ↑Δ[HbO_2_], ↓Δ[HHb], ↑TOI, ↑TD_[HHb]_, ↔ *τ*_[*H*Hb]_^1^	4CWR
Corn and Barstow [166]	HM (7)	Upright cycling	N-acetylcysteine (acute oral supplementation)	↑ CP (232 vs 226 W)	↑GSH, ↑EMG_MPF_ (RF), ↓EMG_RMS_ (VL)	4CWR
Dekerle et al. [56]	HM (5) HF (6)	Upright cycling	Hypoxia (FiO_2_ = 0.15)	↓ CP (190 vs 220 W)	↓SaO_2_	3–4CWR
Valli et al. [61]	HM (4) HF (2)	Upright cycling	Hypoxia (altitude = 5050 m)	↑ CP (123 vs 81 W)	↓$\dot{V}$O2_peak_, ↓blood [lactate], ↓SaO_2_, ↓O_2_ pulse	3 CWR
Broxterman et al. [73]	HM (8)	Handgrip	Duty cycle (50% vs 20%)	↓ CP (3.9 vs 5.1 W)	↓Q̇_BA_, ↑iEMG, ↓EMG_MPF_, ↓m$\dot{V}$O_2_, ↔ [THb], ↓end-exercise [HHb] ^2^	3–4CWR
Mueller et al. [167]	ETM (11)	Upright cycling	Resistance + vibration training (8 weeks)	↑ CP (296 vs 286 W)	↑Capillary:fibre, ↑thigh LBM, ↑MyHC1 and ↑MyHC2 CSA, ↔ SDH	4CWR
Broxterman et al. [168]*	ETM (5) ATM (5)	Upright cycling	Cadence (100 rpm vs 60 rpm)	↓ CP (196 vs 214 W)		4 CWR
Black et al. [169]	HM (10)	Upright cycling	Pacing (self vs constant load)	↑ CP (265 vs 250 W)	↓${\mathrm{MRT}}_{\dot{V}\mathrm{O}2}$, ↑VO_2_ in first 60 s	3–4TT/3–4CWR
Broxterman et al. [75]	HM (6)	Handgrip	Blood flow occlusion	↓ CP (-0.7 vs 4.1 W)	↓EMG_RMS_, ↑[HHb], ↓[HbO_2_], ↓[THb]^2^	4CWR
Parker-Simpson et al. [60]	HF (13)	Upright cycling	Hypoxia (FiO2 = 0.13)	↓ CP (132 vs 175 W) ↓ EP (134 vs 172 W)	↓$\dot{V}$O2_max_	5CWR and 3MT
Deb et al. [170]	ETM (11)	Upright cycling	Hypoxia (FiO_2_ = 0.145) ± sodium bicarbonate	↓ CP (265 vs 263 vs 301 W)	↓SaO_2_	3MT
Goulding et al. [114]	HM (10)	Supine cycling	Heavy priming exercise	↑ CP (185 vs 177 W)	↓${\tau }_{\dot{V}\mathrm{O}2}$, ↔ $\dot{V}$O_2max_, ↑[HbO_2_], ↑*τ*_[HHb]_^1^	4CWR
Townsend et al. [63]	ETM (9)	Upright cycling	Hypoxia (FiO_2_ = 0.18, 0.159, 0.14, 0.123)	↓ CP (257, 235, 218, 196 vs 270 W)		3TT
Clark et al. [171]	ETM (6)	Upright cycling	2 h heavy exercise	↓ CP (282 vs 306 W)		3MT
Goulding et al. [117]	HM (8)	Supine cycling	Exercise transition from elevated baseline	↓ CP (132 vs 146 W)	↑${\tau }_{\dot{V}\mathrm{O}2}$, ↔ $\dot{V}$O2_max_, ↔ [HbO_2_], ↑*τ*_[HHb]_, ↓Δ[HHb]/Δ$\dot{V}$O_2_^3^	4CWR
Goulding et al. [130]	HM (7)	Upright cycling	Exercise transition from elevated baseline	↓ CP (203 vs 213 W)	↑ ${\tau }_{\dot{V}\mathrm{O}2}$, ↑ [HbO_2_], ↑ *τ*_[HHb]_, ↓Δ[HHb]/Δ$\dot{V}$O_2_^1^	4CWR
La Monica et al. [62]	HM (21)	Upright arm cycling	Hypoxia (FiO_2_ = 0.14)	↓ CP (85 vs 90 W)	↓$\dot{V}$O_2peak_	4CWR
Mitchell et al. [86]	ETM (21)	Upright cycling	SIT, SIT + blood flow restriction (4 weeks)	↑ CP (302, 302 vs 292 W)	↑$\dot{V}$O_2peak_, ↔ capillarity, ↔ mitochondrial protein content	3-5CWR
Clark et al. [172]	HM (14)	Upright cycling	2 h heavy exercise	↓ CP (CWR: 256, EP: 256 vs EP: 287 W)	↓Muscle [glycogen], ↔ $\dot{V}$O_2peak_	4CWR and 3MT
Clark et al. [173]	ETM (16)	Upright cycling	2 h heavy exercise	↓ CP (236 vs 260 W)	↓Muscle [glycogen], ↔ $\dot{V}$O_2peak_	3MT
Goulding et al. [64]	HM (8)	Supine cycling	Hyperoxia (FiO_2_ = 0.5)	↑ CP (148 vs 134 W)	↑$\dot{V}$O_2max_, ↓${\tau }_{\dot{V}\mathrm{O}2}$, ↑[HbO_2_], ↔ *τ*_[HHb]_^3^	4CWR
Morgan et al. [152]	HM (16)	Upright cycling	Acetaminophen (acute oral supplementation)	↑ CP (297 vs 288 W)	↑EMG_RMS_, ↔ $\dot{V}$O2_peak_	3MT
Waldron et al. [174]	HM (12)	Upright cycling	Taurine (acute oral supplementation)	↑ CP (212 vs 197 W)	↑Post-exercise blood [lactate]	3MT
Goulding et al. [65]	HM (9)	Upright cycling	Hyperoxia (FiO_2_ = 0.5)	↑ CP (216 vs 197 W)	↑$\dot{V}$O_2max_, ↔ ${\tau }_{\dot{V}\mathrm{O}2}$ ↑PetO_2_, ↑[HbO2], ↓[HHb], ↔ *τ*_[HHb]_^3^	4CWR
Goulding et al. [131]	T1DM (7)	Upright cycling	Heavy priming exercise	↑ CP (161 vs 149 W)	↓${\tau }_{\dot{V}\mathrm{O}2}$, ↔ $\dot{V}$O_2max_, ↔ [HbO_2_], ↓ *τ*_[HHb]_^1^	4CWR
Karabiyik et al. [175]	TM (32)	Upright cycling	SIT (4 weeks) ± hypoxia (FiO_2_ = 0.135)	↑ CP (200 vs 170 W)^#^	↑Post-ramp blood [lactate], ↔ $\dot{V}$O_2peak_	3MT
Collins et al. [176]	HM (5) HF (6)	Upright cycling	Endurance training (8 weeks)	↑ CP (161 vs 140 W)	↑$\dot{V}$O_2max_	3–6CWR
Collins et al. [176]	HM (6) HF (5)	Upright cycling	HIIT (8 weeks)	↑ CP (176 vs 140 W)	↑$\dot{V}$O_2max_	3–6CWR

Study: *latter publication uses a sub-set of data taken from the former publication

Population: *AT* anaerobically trained, *COPD* chronic obstructive pulmonary disease, *ET* endurance trained, *F* female, *M* male, *H* healthy, *n* number of participants, *T1D* type 1 diabetes

Intervention: *FiO*_*2*_ fraction of inspired O_2,_ *HIIT* high-intensity interval training, *rpm* revolutions per minute, *SIT* sprint interval training, ± with and without

Effect on CP: ↑ increased, ↓ decreased, ^#^values for CP estimated from visual inspection of figures

Physiological effects of intervention (all factors considered for chronic interventions, only those factors measured during the determination of CP considered for acute interventions): ^1^ [HHb], [THb], [HbO_2_] determined via near infrared spectroscopy on the VL, ^2^[HHb], [THb], [HbO_2_] determined via near infrared spectroscopy on the flexor digitorum superficialis, ^3^[HHb], [THb], [HbO_2_] determined via near infrared spectroscopy on the VL and RF, *τ*_*[HHb]*_ time constant of [HHb] kinetics, ${\tau }_{\dot{V}\mathrm{O}2}$ time constant of $\dot{V}$O_2_ kinetics, *ADP* adenosine diphosphate, *CSA* cross-sectional area, *EMG*_*MPF*_ electromyography median power frequency, *EMG*_*RMS*_ electromyography root mean squared, *GET* gas exchange threshold, *HbO*_*2*_ oxygenated haemoglobin, *HHb* deoxygenated haemoglobin, *iEMG* integrated electromyography, *La* lactate, *LBM* lean body mass, *LT* lactate threshold, ${\mathrm{MRT}}_{\dot{V}\mathrm{O}2}$ mean response time of $\dot{V}$O_2_, *MVC* maximal voluntary contraction, *m*$\dot{V}$*O*_*2*_ muscle $\dot{V}$*O*_2_ estimated via combined near infrared spectroscopy and doppler ultrasound, *MyHC1* myosin heavy chain 1, *MyHC2* myosin heavy chain 2, *PCr* phosphocreatine, *PetO*_*2*_ end-tidal pressure of O_2_, *Pi* inorganic phosphate, *Q̇*_*BA*_ brachial artery blood flow, *RF* rectus femoris, *SaO*_*2*_ arterial oxygen saturation, *SDH* succinate dehydrogenase, *THb* total haemoglobin, $\dot{V}$*e*_*peak*_ highest ventilation measured, *VL* vastus lateralis, $\dot{V}$*O*_*2*_ rate of oxygen uptake, *VO*_*2*_ total oxygen consumed, $\dot{V}$*O*_*2max*_ maximal $\dot{V}$O_2_ recorded following verification from additional trials > CP, $\dot{V}$*O*_*2peak*_ highest $\dot{V}$O_2_ recorded but not verified with additional tests > CP, ↔ unchanged

CP determination method: *3MT* 3-min all-out test, *nCWR* number of constant work-rate trials, *nTT* number of time trials

**Table 2:** Summary of studies demonstrating physiological or performance factors that correlate with critical power (CP)

Study	Population	Mode	Correlation details	CP determination method
Neder et al. [34]	HM (10) and COPDM (8)	Upright cycling	CP (W) correlated with ${\mathrm{MRT}}_{\dot{V}\mathrm{O}2}$ (s) during severe-intensity exercise in MH (*r* = − 0.65) but not MCOPD	4CWR
Murgatroyd et al. [14]	HM (14)	Upright cycling	CP (W) correlated with ${\tau }_{\dot{V}\mathrm{O}2}$ (s) during severe-intensity exercise (*r* = − 0.95)	4CWR
Black et al. [13]	ETM (10)	Upright cycling	CP (W) correlated with 10-mile TT performance (min) (r = − 0.83)	3MT
Vanhatalo et al. [31]	HM (4) HF (4)	Upright cycling	CP (W) correlated with %type 1 fibre (*r* = 0.67)	3MT
Goulding et al. [114]	HM (10)	Upright cycling	CP (W kg^−1^) correlated with ${\tau }_{\dot{V}\mathrm{O}2}$ (s) during heavy-intensity exercise (*r* = − 0.80)	4CWR
Byrd et al. [177]	ATM (15)	Upright cycling	CP (W) correlated with LBM (kg) (*r* = 0.59)	3MT
Goulding et al. [130]	HM (7)	Upright cycling	CP (W kg^−1^) correlated with ${\tau }_{\dot{V}\mathrm{O}2}$ (s) during moderate-intensity exercise (*r* = − 0.95)	4CWR
Mitchell et al. [86]	ETM (14)	Upright cycling	CP (W) correlated with: %type 1 fibre (*r* = 0.79), no. of capillary contacts in type 1 (*r* = 0.94) and type 2 (*r* = 0.68) fibres, capillary:fibre (*r* = 0.88)	3–4CWR
Goulding et al. [131]	HM (9)	Upright cycling	CP (W kg^−1^) correlated with ${\tau }_{\dot{V}\mathrm{O}2}$ (s) during moderate-intensity exercise (*r* = − 0.92)	4CWR
Smyth and Muniz-Pamares [28]	HM and HF (31,190)	Running	CS (m s^−1^) correlated with marathon time (min) (*r* = − 0.83)	3TT
Collins et al. [176]	HM (11) HF (11)	Upright cycling	CP (W) correlated with: $\dot{V}$O_2max_ (mL min^−1^) (*r* = 0.96), P_peak_ (W) (*r* = 0.97), *P*_max_ (W) (*r* = 0.84), Leg LBM (kg)) (*r* = 0.81) CP (W kg^−1^) correlated with: $\dot{V}$O_2max_ (mL min^−1^ kg^−1^) (*r* = 0.85), *P*_peak_ (W kg^−1^) (*r* = 0.89), *P*_max_ (W kg^−1^) (*r* = 0.52)	3–6CWR

Population: *COPD* chronic obstructive pulmonary disease, *ET* endurance trained, *F* female, *M* male, *H* healthy, *n* number of participants

Correlation details: ${\tau }_{\dot{V}\mathrm{O}2}$ time constant of $\dot{V}$O_2_ kinetics, *LBM* lean body mass, ${\mathrm{MRT}}_{\dot{V}\mathrm{O}2}$ mean response time of $\dot{V}$O_2,_ *P*_*max*_ highest power attained during a 30-s all-out exercise test, *P*_*peak*_ highest power attained during an incremental exercise test, *TT* time trial, $\dot{V}$*O*_*2max*_ maximal $\dot{V}$O_2_ recorded following verification from additional trials > CP

CP determination method: *3MT* 3-min all-out test, *nCWR* number of constant work-rate trials, *nTT* number of time trials

## Interaction of Factors Determining CP

**Fig. 2:**
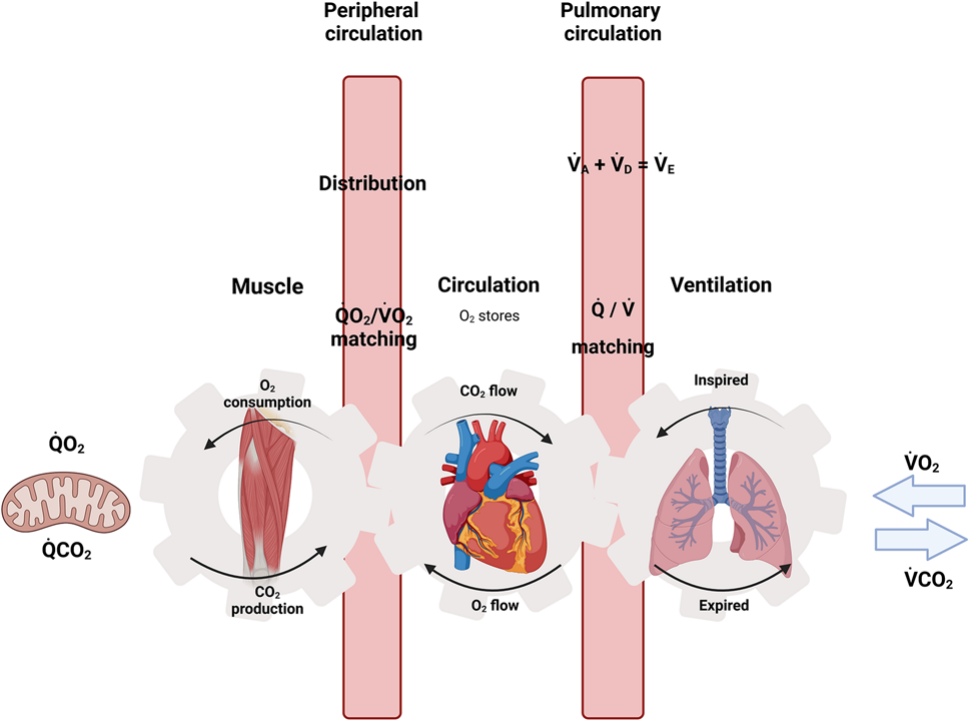
Schematic illustrating an adaptation of Wasserman’s classic “Gears” diagram. It demonstrates Wasserman’s conception of how the respiratory, cardiovascular and neuromuscular systems conflate to enable exercise to be sustained. O_2_ flows from the atmosphere through the lungs, pulmonary and peripheral circulation to the muscle mitochondria where it is ultimately consumed. CO_2_ produced by the contracting muscle flows along the same pathway in reverse. Muscle work leads to increased cardiac output and redistribution of blood flow, and increased ventilation in response to both the increased metabolism and evolution of CO_2_ from the blood as the result of lactic acid buffering. The efficacy of these processes determines the ability to sustain muscular exercise. These concepts are reconsidered in this review within the context of critical power. This figure was created with BioRender.com and was exported under a paid subscription. $\dot{Q}$CO_2_ cellular carbon dioxide production, $\dot{Q}$O_2_$/\dot{V}$O_2_ *matching* matching of oxygen delivery to local oxygen consumption, $\dot{Q}$ O_2_ cellular oxygen consumption, $\dot{Q}/\dot{V}$ *matching* matching of ventilation to perfusion, $\dot{V}$_*A*_ alveolar ventilation, $\dot{V}\mathrm{CO}2$ Pulmonary carbon dioxide production, $\dot{V}$_*D*_ dead space ventilation, $\dot{V}$_*E*_ minute ventilation, $\dot{V}$O_2_ pulmonary oxygen consumption. Adapted from Wasserman et al. [156], with permission

That CP represents the threshold intensity above which exercise cannot be sustained in a steady state indicates that it is a parameter of aerobic function. Consequently, it follows that CP may be affected by any step in the O_2_ transport and utilisation cascade, from atmospheric air down to the muscle mitochondria themselves. Specifically, these steps include: (1) transport of atmospheric O_2_ into the blood via pulmonary diffusion; (2) bulk transport of O_2_ to the muscle via convection (i.e. convective O_2_ delivery); (3) diffusion of O_2_ from capillary to muscle mitochondria (i.e. diffusive O_2_ delivery); and (4) the utilisation of O_2_ by the muscle mitochondria (Fig. 2). Whilst the respiratory system may constrain CP in chronic respiratory disease conditions such as COPD [34, 35, 38–42], in most young healthy individuals, the respiratory system appears to be well adapted to ensure a highly efficient and appropriate homeostatic response to high-intensity exercise [43]. Hence, the remainder of this review will focus on the impact of convective and diffusive O_2_ delivery and mitochondrial O_2_ utilisation on CP, downstream of the respiratory system.

### Convective Oxygen Delivery

Convective O_2_ delivery refers to that achieved via bulk movement of O_2_ within the circulation to the exercising muscles. Convective O_2_ delivery ($\dot{Q}$O_2_, L min^−1^) can thus be defined mathematically as the product of cardiac output (CO, L min^−1^) and arterial O_2_ content (Ca_O2_, mL O_2_ 100 mL^−1^):

$${\dot{Q}\mathrm{O}}_{2}=\mathrm{CO}* {\mathrm{Ca}}_{\mathrm{O}2},$$

where Ca_O2_ is defined as:

$${\mathrm{Ca}}_{\mathrm{O}2}=\left(1.34*\left[\mathrm{Hb}\right]* {\mathrm{Sa}}_{\mathrm{O}2}*0.01\right)+\left(0.03*{\mathrm{Pa}}_{\mathrm{O}2}\right),$$

where 1.34 is Hüfner’s constant describing the maximum O_2_ carrying capacity per gram of haemoglobin (mL O_2_ g^−1^ Hb), [Hb] is haemoglobin concentration (g dL^−1^), SaO_2_ is the arterial saturation of Hb, 0.03 is the solubility coefficient of O_2_ at body temperature (mL O_2_ 100 mL^−1^ plasma kPa^−1^) and PaO_2_ is the arterial partial pressure of O_2_ (mmHg). This provides a measure of whole-body convective O_2_ delivery. However, the O_2_ flux to each portion of the exercising muscles is not uniform but varies according to regional metabolic demands, vascular control and fibre type [44–46].

A convenient means by which to experimentally alter Ca_O2_, and hence, convective O_2_ delivery, is by varying the fraction of inspired O_2_ (FiO_2_). Although hypoxia-induced vasodilatation [47] and hyperoxia-induced vasoconstriction [48] often influence blood flow thereby helping to normalise muscle O_2_ delivery during exercise, many studies that have quantified skeletal muscle O_2_ delivery under these conditions have demonstrated that hyperoxia can enhance and hypoxia can impair skeletal muscle O_2_ delivery during exercise, respectively [49–54], thereby impacting upon intra-myocyte *P*O_2_ (*P*O_2im_) [55]. Indeed, the early work of Moritani et al. [21] showed that, in a limited sample of two participants, inspiration of a hypoxic gas mixture (FiO_2_ 0.09) resulted in a reduced CP compared with normoxia (i.e. FiO_2_ 0.21; hypoxia: 106 ± 6 W, vs normoxia: 214 ± 4 W). Under conditions of more moderate hypoxia (FiO_2_ 0.15) and in a larger sample of 11 subjects, Dekerle et al. [56] found that CP was reduced by 30 W in hypoxia compared with normoxia, consequent to a reduction in arterial O_2_ saturation of 12%. Notably, in this latter study, the percentage decrement in CP between hypoxia and normoxia was correlated with $\dot{V}$O_2max_ in normoxia, suggesting that those with the greatest $\dot{V}$O_2max_ values were better able to offset the reductions in convective O_2_ delivery brought about via hypoxia. It is not known if such a protective effect remains in highly trained athletes where pulmonary limitations to high-intensity exercise are more likely [57, 58], causing reductions in arterial saturation and $\dot{V}$ O_2max_ even at modest simulated altitudes [59]. Similarly, however, Simpson et al. [60] reported a reduction in CP of 43 W using an FiO_2_ of 0.13, a finding that was consistent when CP was determined either via the conventional constant-load prediction trial method or via a 3-min all-out test. Moreover, Valli et al. [61] demonstrated that at an altitude of 5050 m (equivalent FiO_2_ ~ 0.11) CP was reduced by 42 W. In all of these studies, SaO_2_ was reduced either at rest or during exercise in hypoxia, providing indirect evidence that hypoxia impaired convective O_2_ delivery that contributed to the reduced CP in each study. These findings were subsequently extended to arm cycle ergometry by La Monica et al. [62], who demonstrated that arm CP was reduced by 5 W in moderate (FiO2 0.14) normobaric hypoxia (~ 6% of normoxic CP). Whilst the magnitude of the effect of hypoxia on CP in these studies varied with the fitness of the participants (see, for example, Dekerle et al. [56]), Townsend et al. [63] demonstrated a progressive reduction in CP with decreasing FiO_2_. Hence, the extant literature is unanimously consistent with the notion that reductions in FiO_2_ (and by extension, convective O_2_ delivery) reduce CP.

The consistency of the effects of hyperoxia on CP are similar to those of hypoxia. This was first demonstrated by Vanhatalo et al. [37], who assessed the impact of an FiO_2_ of 0.7 on CP utilising a single-leg knee-extension exercise model. These authors showed that CP was increased in hyperoxia compared with normoxia, with a concomitant increase in muscle oxygenation (as determined via near-infrared spectroscopy [NIRS]). The increase in CP was accompanied by a slower rate of change in muscle [PCr], [ADP], [Pi] and pH. Subsequently, these findings for small-muscle mass exercise were confirmed for large-muscle mass exercise by Goulding et al. [64, 65]. Specifically, a hyperoxic inspirate (FiO_2_ of 0.5) resulted in increases in end-tidal *P*O_2_ (and, therefore, alveolar *P*O_2_) and muscle oxygenation determined via NIRS both at rest and during exercise [64, 65]. As a result, CP was enhanced during cycle exercise in hyperoxia versus normoxia in both the supine [64] and upright [65] body positions, with the magnitude of improvement being ~ 10% in both studies. Hence, studies have consistently shown that CP is sensitive to both increased [37, 64, 65] and decreased [21, 56, 60–62] FiO_2_.

Another experimental intervention that has yielded insights into the dependency of CP on convective O_2_ delivery is via manipulations in the muscle contraction duty cycle. Muscle contraction, particularly during small-muscle mass exercise where compressive forces can be high, increases intramuscular pressure, compresses blood vessels, increases impedance to flow and may cause temporary blood flow occlusion [66–69]. Hence, the muscular contraction cycle yields rhythmic alterations in intramuscular pressure, and hence blood flow, with the majority of flow occurring during the relaxation phase of contraction, [69–72]. Utilising a small-muscle mass handgrip exercise, Broxterman et al. [73] directly tested the hypothesis that alterations in the duty cycle would cause concomitant alterations in convective O_2_ delivery, and hence CP, by measuring brachial artery blood flow via Doppler ultrasound during exercise with a 20% and 50% duty cycle (i.e. muscle contraction comprised 20 and 50%, respectively, of the total contraction-relaxation cycle). Brachial artery blood flow, and thus, convective O_2_ delivery, was greater in the 20% duty cycle when compared with the 50% duty cycle, with a concomitant increase in CP [73].

In extending the principle of altering convective O_2_ delivery to observe its effect on CP, Broxterman et al. [74, 75] showed that during blood flow occlusion (which constrains O_2_ delivery to zero), CP was reduced to a negative value. Whilst a negative CP appears implausible, this finding demonstrates a reliance of CP on convective O_2_ delivery as there is no sustainable rate of oxidative metabolism without blood flow. Resting (i.e. 0 W) occlusion results in progressive depletion of [PCr] and muscle/capillary O_2_ stores [76, 77], a feature consistent with non-steady-state conditions [15]. Accordingly, the magnitude of the negative CP during blood flow occlusion would be expected to be proportional to the resting metabolic rate, and as such is entirely plausible.

These findings were recently extended by Hammer et al. [78] where critical force (CF) was estimated during the final minute of repeated handgrip maximum voluntary contraction (MVC) efforts over a 5-min duration. Under free-flowing conditions without occlusion, force progressively declined with time during the test until a plateau was reached in the final minute of the test, termed CF [78]. With muscle occlusion, however, force continuously declined with time, i.e. there was no plateau in force at the end exercise [78]. Following subsequent reperfusion, force was able to recover to a level not significantly different from CF determined under free-flowing conditions [78]. These authors also demonstrated that up to and including CF, end-exercise limb blood flow values were linearly related to the constant-force requirements of each task [79, 80]. However, during exercise slightly above CF, end-exercise brachial artery blood flow demonstrated a plateau, being no different from the blood flow values obtained during exercise at CF [79]. These findings were subsequently extended to large muscle mass, whole-body exercise by the same authors [80]. Specifically, leg blood flow and limb vascular conductance were determined using Doppler ultrasound and calibrated finger plethysmography during exercise above and below CP [80]. Post-exercise increases in limb vascular conductance and leg blood flow post-exercise were observed following supra-CP but not sub-CP exercise [80]. The data of Hammer et al. [79, 80] are in contrast to observations in the running rat [25] and from upright, incremental, large muscle mass exercise in humans [52, 81] showing increases in limb blood flow up to $\dot{V}$O_2max_. Nevertheless, these findings raise the intriguing possibility that in certain contexts, CF/CP represents a threshold in relative muscular force that limits skeletal muscle perfusion during exercise. Moreover, the extant literature appears to be unanimously consistent with CP being determined, at least in part, by mechanisms related to convective O_2_ delivery.

### Diffusive Oxygen Transport

Diffusive O_2_ transport refers to the diffusive movement of O_2_ from the capillaries to the muscle mitochondria where O_2_ serves as the final electron acceptor for the electron transport system. This process is described mathematically via Fick’s law of diffusion:

$${\dot{V}O}_{2}=D{O}_{2}\left(\Delta P{O}_{2}\right),$$

where $\dot{V}$O_2_ corresponds to the rate of O_2_ flux, *D*O_2_ is the muscle diffusing capacity, and Δ*P*O_2_ is the partial pressure difference between the capillary and intra-myocyte spaces (*P*O_2cap_ and *P*O_2im_, respectively). This relationship dictates that elevations in $\dot{V}$O_2_ must be established via changes in either (1) changes in the driving force for O_2_ diffusion (i.e. Δ*P*O_2_ = *P*O_2cap_ − *P*O_2im_) and/or (2) changes in effective diffusing capacity (i.e. *D*O_2_, determined primarily by the aggregate number of blood cells within capillaries adjacent to the myocyte at any given moment [82, 83]).

Fick’s Law of Diffusion predicts that alterations in FiO_2_ will bring about concomitant alterations in CP via altered O_2_ diffusion in addition to convection. For instance, hypoxia reduces and hyperoxia increases both estimated *P*O_2cap_ [84] and *P*O_2im_ [85]_,_ though to differing extents such that ∆PO_2_ is reduced and increased, respectively. Hence, in the studies reviewed in Sect. 2.1 wherein hypoxia reduced [21, 56, 60–62] and hyperoxia increased CP [37, 64, 65], it is also probable that alterations in the transcapillary driving force for O_2_ flux, and thus diffusive O_2_ delivery, also contributed to the alterations in CP observed therein, likely via the alterations this would be expected to have on *P*O_2im_ [55].

Muscle capillarity is an important influence on *D*O_2_, and thus diffusive O_2_ delivery, as it determines the number of red blood cells adjacent to contracting fibres and thus the surface area available for O_2_ diffusion. Indeed, Mitchell et al. [86] recently demonstrated a striking relationship between CP and skeletal muscle capillary density (*r* = 0.50), capillary-to-fibre ratio (*r* = 0.88) and capillary contacts per type 1 fibre (*r* = 0.94) in a homogenous group of endurance-trained individuals (63.2 ± 4.1 mL kg^−1^ min^−1^, range: 58.7–72.2 mL kg^−1^ min^−1^). These findings indicate that enhancements in diffusive O_2_ flux enable a metabolic steady state to be attained for a greater range of power outputs (i.e. extending the range upwards), thus increasing CP.

Further insight into the role of diffusive factors in determining CP/CF was provided by a series of experiments by Ansdell et al. that compared the power-duration relationship between the sexes during small- [87] and large-muscle mass exercise [88]. It was demonstrated that CF occurred at a greater relative percentage of the MVC in female individuals compared with male individuals during small-muscle mass, intermittent, isometric single-leg knee extension exercise [87]. Conversely, there were no differences observed in the relative percentage of MVC at which CP occurred between male and female individuals during large-muscle mass dynamic cycle exercise [88]. Female individuals have previously been demonstrated to possess a greater degree of capillarity in skeletal muscle and a greater proportion of type I fibres when compared with male individuals [89–91], suggesting a greater capacity for diffusive O_2_ transport. Moreover, during small-muscle mass knee extension exercise, far greater mass-specific rates of blood flow are achieved when compared with cycle exercise, and hence, diffusive rather than convective factors constrain O_2_ transport to muscle mitochondria [52, 81, 92–97]. These authors [87, 88] consequently interpreted their findings to indicate that during single-limb exercise where convective factors are not limiting, the sex difference in CF arises because of a greater skeletal muscle diffusive capacity of female individuals [87, 88]. Conversely, during dynamic cycle exercise where muscle O_2_ delivery is constrained by the central nervous system to prevent a dangerous fall in mean arterial pressure [98], convective O_2_ delivery may be relatively more important in determining CP than muscle diffusive capacity, leading to the lack of a sex difference in this mode of exercise [87, 88].

Utilising measurements of brachial artery blood flow via Doppler ultrasound and NIRS to determine muscle O_2_ extraction, Broxterman et al. [73] were able to estimate muscle $\dot{V}$O_2_ and thereby estimate the contributions of enhanced convective and diffusive O_2_ delivery to the changes in CP they observed between 20 and 50% duty cycles (discussed in *Convective Oxygen Delivery*). These authors demonstrated that the increase in *D*O_2_ in the 20% versus the 50% duty cycle was approximately double the increase in convective O_2_ delivery that occurred between the same trials (i.e. + 69% vs + 34%, respectively), implicating changes in diffusive, rather than convective, O_2_ delivery as being a more important determinant of CP in this situation. These authors suggested that the shorter duty cycle would have facilitated higher red blood cell velocity and therefore increased the surface area of the capillary involved in gas exchange (i.e. longitudinal capillary recruitment [99]), thereby enhancing *D*O_2_ and contributing to the increased CP. Interestingly, this observation is also consistent with the suppositions of Ansdell et al. [87, 88] noted above, namely that diffusive factors may be more important for constraining CP during small- versus large-muscle mass exercise. That *D*O_2_ is an independent determinant of CP was recently confirmed by Colburn et al. [100]. Specifically, the vascular ATP-sensitive K^+^ channel inhibitor glibenclamide decreased CS in rats, and this was accompanied by a 25% decrease in *D*O_2_ determined from measurements of skeletal muscle blood flow, arterial O_2_ content, and interstitial and microvascular O_2_ pressures [100]. Collectively, therefore, there is now a growing body of evidence to indicate that CP can be influenced by factors dictating the rate of diffusion of O_2_ from capillaries to mitochondria.

### Oxygen Utilisation

A sentinel parameter defining the skeletal muscle bioenergetics system is the time constant of the fundamental phase of muscle $\dot{V}$O_2_ kinetics (i.e. ${\tau }_{\dot{V}\mathrm{O}2}$), which is reflective of the time taken to attain 63% of the $\dot{V}$O_2_ amplitude in response to a change in metabolic demand [101–104], and is closely reflected by the pulmonary ${\tau }_{\dot{V}\mathrm{O}2}$ [103]. Pulmonary ${\tau }_{\dot{V}\mathrm{O}2}$ is therefore a highly convenient assay of the time course of changes in oxidative phosphorylation that occur at the onset of exercise or during changes in the metabolic rate. At the onset of exercise, therefore, the delayed response of pulmonary and muscle $\dot{V}$O_2_ kinetics that is encapsulated by the parameter ${\tau }_{\dot{V}\mathrm{O}2}$ necessitates an energy deficit that must be met via a reduction in O_2_ stores and an increased rate of substrate-level phosphorylation [103, 105, 106]. This “O_2_ deficit” is a function of ${\tau }_{\dot{V}\mathrm{O}2}$ and the steady-state increment $\dot{V}$O_2_ [105], at least for work rates where a steady state is rapidly attained. The magnitude of this O_2_ deficit at exercise onset is critical, as it determines (1) the degree of reliance on non-oxidative sources of energy provision (i.e. depletion of [PCr] and [glycogen] and consequent accumulation of [L^−^] and [H^+^]), (2) the magnitude of metabolic perturbation incurred during the rest-to-work transition (i.e. Δ[PCr], Δ[ADP], Δ[Pi], extracellular [K^+^] accumulation, loss of sarcoplasmic Ca^2+^ release and sensitivity), (3) the extent of fatigue induction sustained and (4) the loss of skeletal muscle efficiency induced during the rest-to-exercise transition [8, 10, 14, 101, 102, 104, 107–110]. $\dot{V}$O_2_ kinetics would therefore appear to be central in setting the tolerability of exercise. Indeed, very low ${\tau }_{\dot{V}\mathrm{O}2}$ values (i.e. fast $\dot{V}$O_2_ kinetics) are observed in endurance athletes [111] and trained individuals [112], whereas very large ${\tau }_{\dot{V}\mathrm{O}2}$ values (i.e. slow $\dot{V}$O_2_ kinetics) are observed in the elderly [113] and chronically ill [102]. However, until relatively recently, an independent role for ${\tau }_{\dot{V}\mathrm{O}2}$ in determining CP had not been considered.

Murgatroyd et al. [14] characterised relationships between ${\tau }_{\dot{V}\mathrm{O}2}$ and CP by normalising exercise intensity across individuals such that the tolerable duration of exercise was uniform (6 min). They demonstrated a strong inverse correlation between ${\tau }_{\dot{V}\mathrm{O}2}$ and CP (*r* = 0.95), consistent with the notion that ${\tau }_{\dot{V}\mathrm{O}2}$ has an independent role in determining CP. Moreover, when this analysis was extended across human populations spanning the extremes of aerobic function (i.e. healthy young trained individuals, young inactive individuals, healthy elderly individuals and patients with COPD), the relationship between ${\tau }_{\dot{V}\mathrm{O}2}$ and CP was strong, inverse and linear [104]. These authors interpreted this relationship causally: by minimising the reliance on substrate-level phosphorylation, and hence the accumulation of fatigue-related metabolites during the transition, a lower ${\tau }_{\dot{V}\mathrm{O}2}$ (i.e. faster $\dot{V}$O_2_ kinetics) allows a higher power production to be achieved for a given magnitude of O_2_ deficit accumulation. Critical power represents the upper limit of the metabolic steady state, and by extension also signifies the upper limit of an O_2_ deficit below which muscle fatigue, reduction in work efficiency and the O_2_ deficit itself will stabilise. All else being equal, therefore, faster $\dot{V}$O_2_ kinetics will result in a higher CP. However, despite the strong rationale and cross-sectional evidence supporting a mechanistic link between ${\tau }_{\dot{V}\mathrm{O}2}$ and CP, until recently, this hypothesis had not received direct experimental scrutiny.

In the first of a series of studies examining the purported determining effect of ${\tau }_{\dot{V}\mathrm{O}2}$ on CP, Goulding et al. [114] examined the influence of prior heavy (“priming”) exercise on pulmonary $\dot{V}$O_2_ kinetics and CP during supine and upright cycling. A prior bout of priming exercise does not speed $\dot{V}$O_2_ kinetics (i.e. reduce ${\tau }_{\dot{V}\mathrm{O}2}$) during upright cycle exercise in young healthy individuals. However, during exercise in the supine position, muscle perfusion pressure is impaired and ${\tau }_{\dot{V}\mathrm{O}2}$ becomes O_2_ delivery dependent [114–120]. Hence, in a young healthy population, prior heavy exercise (which enhances muscle O_2_ delivery, [115, 121, 122]) would be expected to reduce ${\tau }_{\dot{V}\mathrm{O}2}$ during supine but not upright cycling. Accordingly, should ${\tau }_{\dot{V}\mathrm{O}2}$ exert a determining effect on CP, an increase in CP during supine, but not upright, exercise would be observed following priming exercise as compared with control conditions. It was demonstrated that when priming exercise was conducted in the supine position, ${\tau }_{\dot{V}\mathrm{O}2}$ was indeed reduced and CP concomitantly increased, whereas during upright exercise, both ${\tau }_{\dot{V}\mathrm{O}2}$ and CP were unaffected [114]. These findings therefore provided the first experimental evidence that ${\tau }_{\dot{V}\mathrm{O}2}$ is mechanistically related to CP.

Because of the nature of the priming intervention utilised in this first study [114], however, it was not possible to separate any independent effect of a reduced ${\tau }_{\dot{V}\mathrm{O}2}$ (i.e. slowed $\dot{V}$O_2_ kinetics) on CP from that of an improved O_2_ availability as a consequence of the priming exercise. Indeed, the strong correlation observed between ${\tau }_{\dot{V}\mathrm{O}2}$ and CP for upright exercise was absent for supine exercise [114]. Hence, it remained plausible that, at least in supine exercise, other physiological factors, such as muscle O_2_ availability, and its distribution relative to $\dot{V}$O_2_, determine CP, with the concomitant improvements in ${\tau }_{\dot{V}\mathrm{O}2}$ and CP being an artefact of shared physiological determinants, without any dependence of CP on ${\tau }_{\dot{V}\mathrm{O}2}$ per se. Hence, confirmation or refutation of the hypothesis that ${\tau }_{\dot{V}\mathrm{O}2}$ is an independent determinant of CP required an intervention that could alter ${\tau }_{\dot{V}\mathrm{O}2}$ without any concomitant alterations in muscle O_2_ delivery, such that the independent effect of ${\tau }_{\dot{V}\mathrm{O}2}$ on CP could be observed. When exercise is initiated from an elevated baseline work rate, ${\tau }_{\dot{V}\mathrm{O}2}$ is greater than when compared with work initiated from a baseline of unloaded cycling [123–126]. Importantly, this slowing of the $\dot{V}$O_2_ kinetics appears to occur independently of any alterations in O_2_ availability [127–129].

Hence, we conducted two further studies that assessed the influence of exercise initiated from an elevated baseline work rate on ${\tau }_{\dot{V}\mathrm{O}2}$ and CP in the upright [130] and supine [117] positions. In both of these studies, ${\tau }_{\dot{V}\mathrm{O}2}$ was greater (i.e. $\dot{V}$O_2_ kinetics was slower) and CP was correspondingly reduced during work-to-work exercise compared with when exercise was initiated from a baseline of unloaded cycling [117, 130]. Crucially, indicators of O_2_ availability determined via NIRS were either improved [130] or unchanged [117] during work initiated from an elevated baseline, suggesting that the slowing of $\dot{V}$O_2*p*_ kinetics brought about by this intervention was wholly independent of changes in microvascular O_2_ availability. Taken together, these findings therefore demonstrate an independent effect of ${\tau }_{\dot{V}\mathrm{O}2}$ on CP [130], and that this effect persisted even in situations where O_2_ delivery is substantially impaired [117].

The determining effect of ${\tau }_{\dot{V}\mathrm{O}2}$ on CP observed in healthy populations [64, 114, 117, 130] was later confirmed in a study that assessed the impact of priming exercise on $\dot{V}$O_2_ kinetics and CP in a population of individuals with type 1 diabetes mellitus [131]. In this population, priming exercise speeded $\dot{V}$O_2_ kinetics and increased CP during subsequent severe-intensity cycle exercise. Notably, these effects were accompanied by a concomitant speeding of muscle deoxygenation kinetics determined via NIRS [131]. As the muscle deoxygenation signal derived via NIRS represents the relative balance between O_2_ delivery and utilisation within the interrogated region, a relative speeding of muscle deoxygenation kinetics suggests that the effects of priming exercise on ${\tau }_{\dot{V}\mathrm{O}2}$ were predominantly due to an upregulation of otherwise impaired intracellular mechanisms of mitochondrial O_2_ utilisation, rather than O_2_ delivery [131]. Taken together, therefore, substantial recent evidence has accumulated to demonstrate that rates of intracellular O_2_ utilisation at the onset of exercise, encapsulated by ${\tau }_{\dot{V}\mathrm{O}2}$, can influence CP independently of factors related to mitochondrial O_2_ provision.

## Interaction of Factors Determining CP

The studies of Goulding et al. [8, 64, 65, 114, 117, 130, 131] provide convincing evidence that ${\tau }_{\dot{V}\mathrm{O}2}$ is an independent determinant of CP. As reviewed above, there is also evidence for an independent determining role of convective and diffusive O_2_ delivery in influencing CP. That each of ${\tau }_{\dot{V}\mathrm{O}2}$_,_ convective and diffusive O_2_ delivery has an independent role in determining CP is evinced by the fact that each can alter CP without a concomitant change in the other.

**Fig. 3:**
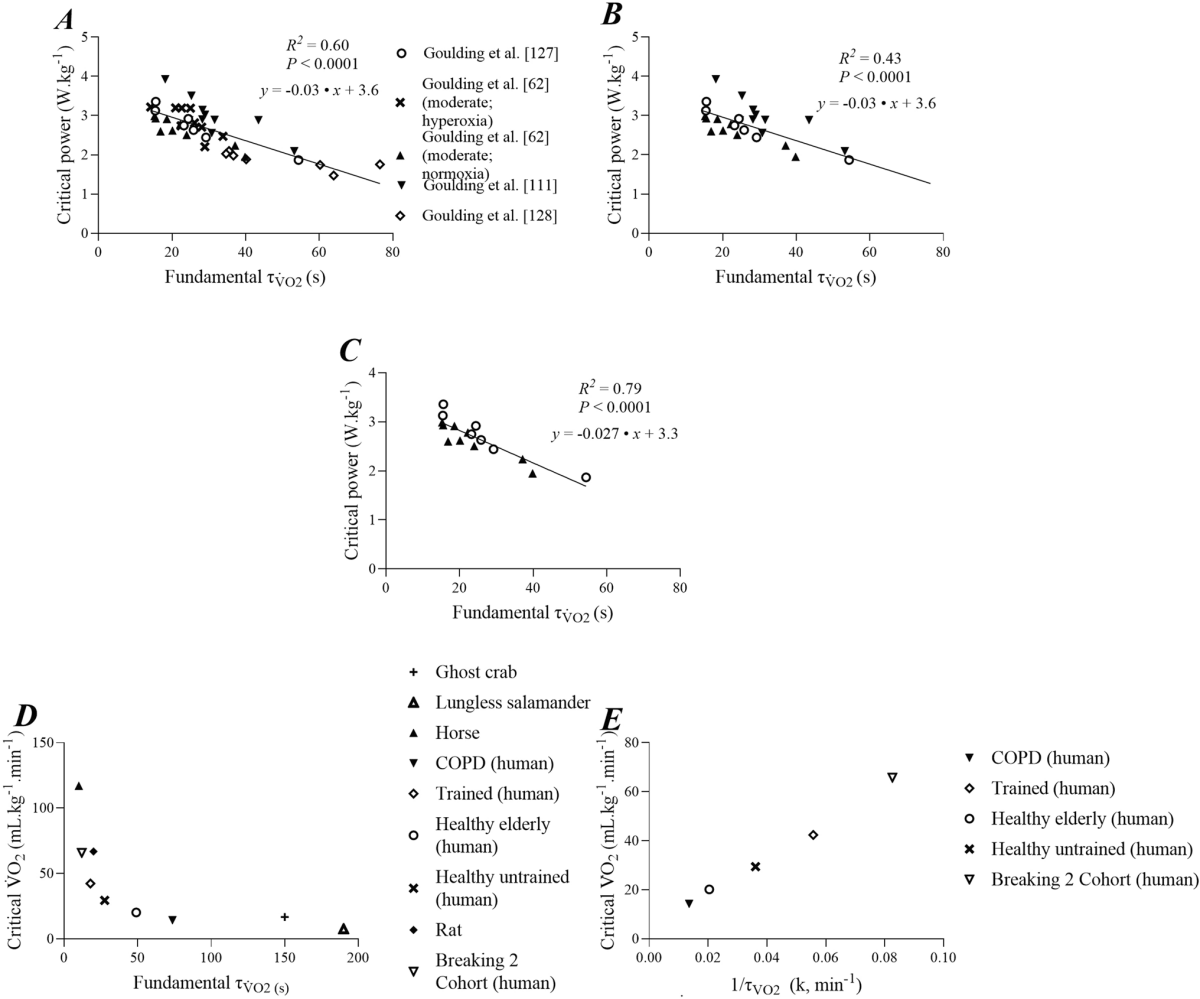
Panels **A–C** show the relationship between the fundamental phase time constant of pulmonary oxygen uptake kinetics (${\tau }_{\dot{V}\mathrm{O}2}$) and critical power normalized by body mass across a series of four experiments performed by Goulding et al. [65, 114, 130, 131]. Panel **A** displays all conditions from these studies in which ${\tau }_{\dot{V}\mathrm{O}2}$ was characterised with a high degree of confidence, including both moderate- and heavy-intensity exercise, normoxia and hyperoxia (fraction of inspired O_2_ = 0.5), and in patients with type 1 diabetes mellitus. Panel **B** displays the same relationship with removal of data points where ${\tau }_{\dot{V}\mathrm{O}2}$ was characterised in hyperoxic conditions and in type 1 diabetes (see “Sect. 2” for discussion). Panel **C** displays the relationship when only normoxic moderate-intensity exercise transitions in healthy participants are utilised. Note the increase in the *R*^*2*^ value as the conditions become more uniform with respect to exercise intensity, population and fraction of inspired O_2_. Panel **D** shows the relationship between ${\tau }_{\dot{V}\mathrm{O}2}$ and critical $\dot{V}$O_2_ across various human populations; elite athletes [157], young trained, active young, healthy elderly, and patients with chronic obstructive pulmonary disease (COPD) and other species where measurements of ${\tau }_{\dot{V}\mathrm{O}2}$ and critical power have both been conducted (i.e. the thoroughbred racehorse, rat, ghost crab and lungless salamander). The figure is derived from values reported in the literature of 28 papers published between 1982 and 2010; human populations were originally reported by Rossiter [104], with groups which were approximately matched for age, $\dot{V}$O_2max_ and health status. Table S1 of the Electronic Supplementary Material should be consulted for details regarding derivation of critical $\dot{V}$O_2_ in different species. Panel **E** shows human-only data from panel **D** of critical power (CP) [mL kg^−1^ min^−1^] plotted as a function of 1/${\tau }_{\dot{V}\mathrm{O}2}$ (i.e. the rate constant, *k*). There is a notable linear relationship across what can be regarded as the complete range of human fitness, indicating that the relationship between ${\tau }_{\dot{V}\mathrm{O}2}$ and CP is hyperbolic, with previously published linear relationships likely being a function of participant homogeneity, and thus reflecting only a truncated portion of the hyperbolic relationship

The proportion of CP explained by ${\tau }_{\dot{V}\mathrm{O}2}$ has been reported to be as high as 90% in a homogenous participant group where relative exercise intensity was precisely controlled (i.e., a tolerable duration of 6 min across subjects) [14]. Our own data have demonstrated *R*^2^ values of 0.64–0.90 for the relationship between CP and ${\tau }_{\dot{V}\mathrm{O}2}$ during upright exercise [60, 111, 127, 128]. Collation of these data across differing exercise intensity domains and populations, including hyperoxia conditions, yields an *R*^2^ = 0.60 (Fig. 3A; data from [131] previously unpublished), with a slope of ~ 0.03 W kg^−1^ s^−1^. However, this includes data from diseased populations (type 1 diabetes [131]) and hyperoxia [65], both of which might be expected to confound the analysis as the latter may distort the relationship between pulmonary and muscle ${\tau }_{\dot{V}\mathrm{O}2}$ and the former has a slope (0.01 W kg^−1^ s^−1^) significantly different to the healthy populations. Exclusion of diseased and hyperoxic data blunts the strength of the relationship between CP and ${\tau }_{\dot{V}\mathrm{O}2}$ (*R*^2^ = 0.43; Fig. 3B). However, the strength of this relationship increases markedly when only moderate-intensity exercise in healthy participants is considered (*R*^2^ = 0.79; Fig. 3C). The slope of the relationship between ${\tau }_{\dot{V}\mathrm{O}2}$ and CP was preserved across this latter analysis, and taken together, CP appears to be well predicted from ${\tau }_{\dot{V}\mathrm{O}2}$ when the latter is precisely determined for a given relative exercise intensity, varying by ~ 0.03 W kg^−1^ per second change in ${\tau }_{\dot{V}\mathrm{O}2}$. However, and perhaps exemplified by the data from type 1 diabetes [131] and hyperoxia [65], when this relationship is expanded to cover the range of values for ${\tau }_{\dot{V}\mathrm{O}2}$ encountered across the animal kingdom (Fig. 3D), the relationship with CP appears curvilinear, but nevertheless preserved, suggesting a fundamental linkage of CP with muscular bioenergetics across species. Moreover, when the human-only data are considered and the speed of oxygen uptake kinetics expressed as a rate constant (i.e., 1/${\tau }_{\dot{V}\mathrm{O}2}$), the relationship with CP is linear (Fig. 3E). Accordingly, when the scope of human aerobic fitness is considered, the relationship between CP and ${\tau }_{\dot{V}\mathrm{O}2}$ can be considered to be hyperbolic_,_ with previously published linear relationships [14, 104] being an artefact of participant homogeneity. By contrast, only one previous study has titrated the effect of oxygen delivery on CP [63]. Here, the reduction in CP with increasing altitude as a proxy for oxygen delivery was established, simulated by changes to FiO_2_. A non-linear (third-order polynomial) relationship was established with increases in altitude producing progressively larger reductions in CP. Critical power was reduced by 74 W with a 4000-m increase in altitude, though any such relationship will inevitably be impacted by the effect of reductions in FiO_2_ increasing ${\tau }_{\dot{V}\mathrm{O}2}$ (i.e. slowing $\dot{V}$O_2_ kinetics).

**Fig. 4:**
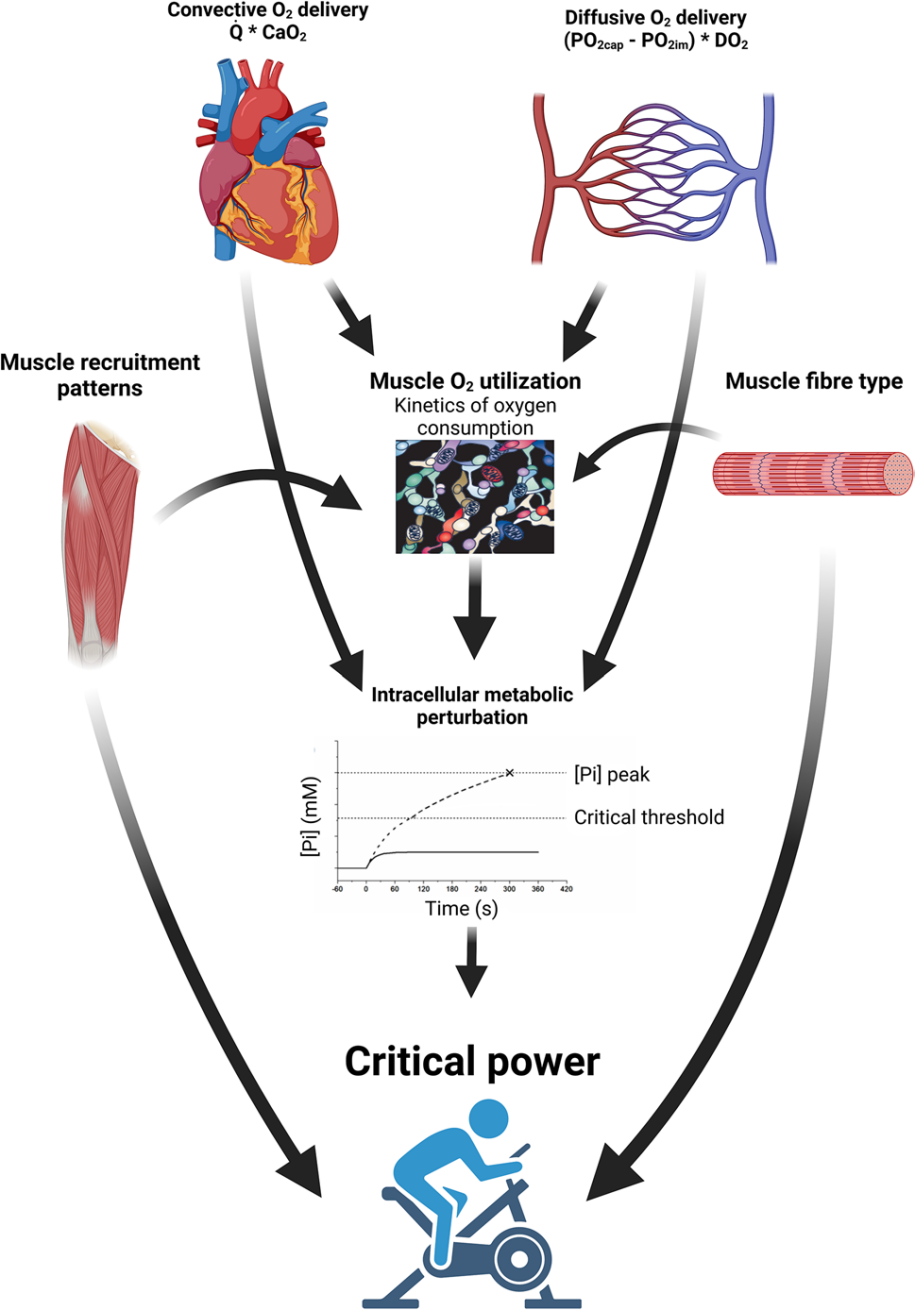
Schematic illustrating each of the factors that has been demonstrated to impact upon critical power. Convective and diffusive O_2_ delivery act in concert with muscle O_2_ utilisation to determine the degree of intracellular metabolic perturbation and fatigue induction incurred during the rest-to-exercise transition. The extent of such metabolic perturbations, in turn, determines whether an exercise bout can be met in a metabolic steady state within a given myocyte. Within a given individual, whether an extant power output is met in a whole-body steady state will depend on the muscle fibre-type composition of the individual, the muscle recruitment patterns employed during the task, and the extent of metabolic derangement and fatigue induction incurred in the recruited fibres during the rest-to-exercise transition. This figure was created with BioRender.com and was exported under a paid subscription. *CaO*_*2*_ arterial oxygen content, *DO*_*2*_ muscle diffusive capacity, *PO*_*2im*_ intra-myocyte O_2_ pressure, *PO*_*2cap*_ capillary O_2_ pressure, $\dot{Q}$, cardiac output

Given the evidence reviewed herein, we therefore propose that each of mitochondrial O_2_ utilisation (encapsulated by the ${\tau }_{\dot{V}\mathrm{O}2}$ parameter), convective and diffusive O_2_ delivery exert independent effects on CP such that intracellular O_2_ utilisation and O_2_ transport interact to determine CP (Fig. 4). Exceptions to this include where pulmonary limitations (e.g. [34]) are dominant factors in limiting exercise tolerance to the extent that they dictate the shape of the power–duration relationship.

The precise mechanisms underpinning such an interaction have not been fully elucidated; however, a starting point is to consider the inexorable loss of intracellular homeostasis, and thus unsustainable rise in O_2_ deficit, during exercise above, but not below, CP. This is accompanied by a mirror-like association between peripheral fatigue [107, 132] and the loss of exercise efficiency [109, 133, 134] that occurs during exercise above CP [135]. Of the factors that accumulate as a result of the O_2_ deficit, [Pi] is a prime candidate for the common denominator between fatigue and efficiency owing to its central role in muscle fatigue and task failure [136]. A recent in silico study by Korzeniewski and Rossiter [10] tested the hypothesis that accumulation of [Pi] during the transition from rest to work could explain both the loss of intracellular homeostasis during supra-CP exercise and the fatigue-related termination of exercise. Using a validated model of the human bioenergetic system, Korzeniewski and Rossiter [10] defined a “critical” (i.e. threshold) [Pi] above which further [Pi] accumulation drove an increase in the requirements for ATP turnover (i.e. an increased ATP cost of muscle contraction) and a “peak” (i.e. limiting) [Pi] at which exercise would cease. The additional ATP turnover driven by [Pi] accumulation resulted in a self-propagating positive feedback loop where additional ATP turnover resulted in increased [Pi], which caused fatigue and additional ATP turnover, until the pre-defined peak [Pi] (and accompanying muscle $\dot{V}$O_2max_) was achieved. By contrast, when [Pi] accumulated below or only marginally above critical [Pi], this positive feedback loop stabilised such that [Pi] did not attain peak values and muscle oxygen uptake attained a steady state. Based on these findings, we therefore recently proposed a model whereby muscle O_2_ consumption kinetics determine CP by dictating the magnitude of O_2_ deficit (and thus [Pi], amongst other factors) accumulated during a given exercise transition [8]. Slow $\dot{V}$O_2_ kinetics begets large intracellular perturbations whereas fast $\dot{V}$O_2_ kinetics engenders smaller intracellular perturbations for a given metabolic rate at exercise onset [102, 104, 110, 137]. Accordingly, more rapid $\dot{V}$O_2_ kinetics will enable a higher exercise intensity before a critical value of [Pi] is breached, thereby increasing critical power, all else being equal. Importantly, simulating alterations in *P*O_2im_ within the computer model of Korzeniewski and Rossiter [10] resulted in the changes in ${\tau }_{\dot{V}\mathrm{O}2}$ and CP predicted by the evidence reviewed in each of the previous sections [10].

Alongside other O_2_ deficit-related factors, that breaching a critical [Pi] results in an inexorable cascade of increasing [Pi], fatigue and ATP turnover is also consistent with the evidence reviewed herein whereby convective and diffusive O_2_ delivery has a determining effect on CP. O_2_ delivery is known to regulate the concentrations of phosphate metabolites at a given metabolic rate, such that when intracellular *P*O_2_ is higher, the intracellular perturbations incurred for [Pi], [PCr] and [ADP] are reduced, whereas the reverse is true when intracellular *P*O_2_ is lower [49, 50, 54, 138]. From these observations, it follows that the aforementioned effects of convective and diffusive O_2_ delivery and intracellular O_2_ utilisation on CP stem from their impact upon the intracellular metabolic state, or more specifically, the rate of ATP turnover at which a critical threshold for [Pi] (which is itself a proxy for a collection of intracellular metabolites reflecting the intracellular state of fatigue) is attained. Hence, faster $\dot{V}$O_2_ kinetics, as well as increased O_2_ delivery, exert their effects on CP via reducing the intracellular metabolic perturbations required to sustain a given rate of ATP turnover, thus enabling a higher power output to be achieved before CP is reached.

## Integration of Mechanisms: Whole Body

This model of CP being an emergent property of the metabolic derangements established at the onset of exercise may provide an explanation for the metabolic bases of CP at the level of a single fibre; however, it does not pretend to be a complete explanation of CP at the integrative whole-body level. This is despite the in silico approach of Korzeniewski and Rossiter [10] being “chimeric”, in that it is built using the data of whole-body and whole-muscle responses of $\dot{V}$O_2_, [PCr], [Pi] and pH and reflecting a variety of muscle fibre types, averaged into a single response. In practice, the exercise transition is undertaken by muscle fibres across the spectrum of function, with differing underlying oxidative phosphorylation activities, each-step activation intensities, convective and diffusive O_2_ supply, and fatigue characteristics [139–141]. Additionally, the location of a given fibre with respect to the skin surface has implications for the relative O_2_ delivery [45, 118–120, 142]. Nevertheless, findings at the whole muscle level are congruent with the notion that metabolic inertia at the onset of exercise determines CP via its effect on the accumulation of Pi and other O_2_ deficit-related metabolites that are implicated in the fatigue process. Type I fibres possess faster $\dot{V}$O_2_ kinetics, better metabolic control, and maintain greater values for capillary and interstitial *P*O_2_ at rest and during contractions [139, 140, 143–150]. Hence, as detailed earlier, in human biopsy studies, the proportion of type I fibres and indices of muscle fibre capillarisation have been shown to be closely associated with CP [31, 86].

Moreover, given that type I fibres maintain greater values for capillary and interstitial *P*O_2_ at rest and during contractions (presumably due to enhanced capillarisation) [139, 140, 143–150], these data are consistent with the present proposal that ${\tau }_{\dot{V}\mathrm{O}2}$, convective and diffusive O_2_ delivery each exert independent interactive determining effects on CP. However, the eventual external outcome of interest from all of these processes, i.e. CP, will also be a function of factors such as (relative) exercising muscle mass, the local musculoskeletal lever system dynamics and co-ordination, the extent of localised fatigue within working muscle groups and motor-unit recruitment. Indeed, that our data demonstrate a significant relationship between ${\tau }_{\dot{V}\mathrm{O}2}$ and CP expressed in W kg^−1^, but not W (data not shown), speaks to the role of exercising muscle mass in the eventual determination of CP. The individual muscles of the quadriceps muscle group have been shown to produce divergent patterns of [PCr] depletion and [Pi] accumulation within distinct muscle regions (72-cm^3^ voxels) during fatiguing (incremental) exercise [151]. Exercising muscle mass may therefore also play a role in the extent of such muscle metabolite heterogeneity, and thus the degree of metabolic perturbation within distinct muscle regions, which in turn contributes towards setting CP.

Morgan and colleagues [152, 153] provided insight into how muscle recruitment patterns may act to determine CP. During repeated intermittent isometric contractions, the ingestion of acetaminophen led to a smaller reduction in torque across 60 MVCs when compared with a placebo [153]. This was associated with a greater preservation of muscle activation with acetaminophen as assessed via electromyography. Subsequently, it was shown that during upright cycle ergometry, acute acetaminophen ingestion increased CP and preserved muscle activity throughout the duration of exercise when compared with a placebo [152]. These findings suggest blunting neuromuscular fatigue development and preserving muscle activation enhances CP, and thus demonstrates the importance of motor unit recruitment profiles.

The interaction between muscle recruitment patterns and muscle O_2_ delivery in determining CP is perhaps most strikingly illustrated by the recent study of Hammer et al. [78], discussed previously (see Sect. 2.1). These authors showed that, following muscle reperfusion, both muscular activity and force production returned to levels not different from those observed under free-flowing conditions [78]. Hence, muscle occlusion constrained muscular recruitment and thus critical force; however, once muscle perfusion was restored to pre-occlusion conditions, both muscle recruitment and force-generating capacity were restored. These findings illustrate that CP represents an intricate balance between muscle O_2_ supply, muscle recruitment patterns and peripheral fatigue development.

To summarise, CP is sensitive to muscle fibre type composition because it is a parameter of aerobic function. Hence, the oxidative characteristics inherent within type I fibres, such as rapid $\dot{V}$O_2_ kinetics, greater rates of blood flow, and higher capillary and interstitial *P*O_2_ values, allow the attainment of high rates of ATP utilisation with minimal derangement of the intracellular metabolic milieu. Therefore, all else being equal, individuals with a relatively greater proportion of type I skeletal muscle fibres will tend to possess greater CP values when compared with individuals of equivalent training status with a greater proportion of type II fibres. Moreover, animal data indicate that CP appears to be a critical threshold for the recruitment of high-order motor units containing a high fraction of type II fibres [25]. Hence, individuals with more type I fibres will attain a relatively greater fraction of their $\dot{V}$O_2_ max before reaching the threshold for progressive recruitment of type II fibres, i.e. CP (as seen in highly trained humans, [111, 154]). Interventions that increase motor unit recruitment are also conducive to high CP values, as a greater number of motor units/muscle fibres performing a given task will lessen the metabolic strain on each individual fibre. Hence, when muscular recruitment is increased, each fibre is able to maintain intramuscular metabolite accumulation below its critical threshold for a wider range of ATP utilisation rates, thus enabling a greater CP, as suggested for the effects of priming by Burnley et al. [155]. Therefore, although there is clearly a role for convective and diffusive O_2_ delivery and intracellular O_2_ utilisation in determining CP, understanding the physiology of CP at the level of integrative physiology is only possible via consideration of how these factors interact with muscle fibre-type composition and recruitment patterns.

## Conclusions

Critical power separates the heavy and severe exercise-intensity domains wherein qualitatively divergent physiological responses are observed, such that CP represents the threshold intensity above which a metabolic steady state cannot be attained during exercise. Hence, CP is fundamental to the understanding of human endurance performance and the causes of exercise limitation in populations where exercise tolerance is impaired. Over the past 15 years or so, evidence has emerged that CP also represents a key threshold for a variety of aspects of physiological system behaviour, such as muscle fibre recruitment, blood flow and vascular control, as well as muscle fatigue. Accordingly, a wide range of evidence has emerged, spanning each step of the oxygen transport pathway, that CP is a fundamental parameter of aerobic function. It has been demonstrated that alterations in delivery of O_2_ to the exercising muscles, via both convection and diffusion, impact upon CP. The rates of O_2_ utilisation during exercise, particularly during the transition from rest to work, also play a key role in determining CP by governing the degree of matching between the rates of ATP utilisation and production. These factors each interact with one another, and via this interaction determine the degree of intracellular metabolic disturbance required to sustain a given power output. How each of these factors interacts to determine CP at the whole-body level will be dependent upon the muscle fibre-type composition and their recruitment patterns during exercise.

## Supplementary Information

Below is the link to the electronic supplementary material.

Supplementary file1 (PDF 84 KB)
