# Supplementary material for: Interaction of Factors Determining Critical Power
Source: Sports Med. 2023 Jan 9;53(3):595–613. doi: 10.1007/s40279-022-01805-w (PMC9935749; doi:10.1007/s40279-022-01805-w)
Supplement: Supplementary file 1 — Supplementary file1 (PDF 84 KB) [file 40279_2022_1805_MOESM1_ESM.pdf]

Table S1

The particular populations reported in Figure 3D are presented in the table below, with associated fundamental phase $\dot{V}O_2$ kinetics time constant ($\tau_{\dot{V}O2}$, s) critical power (W) and estimated critical $\dot{V}O_2$ (ml.kg^{-1}.min^{-1}) values. SD: standard deviation. Superscript value refers to the method by which each critical $\dot{V}O_2$ was calculated. See text below table for details relating to each superscript value.

**Population/ Species**	**Study**	**$\tau_{\dot{V}O2}$ (s)**	**Critical power (W)**	**Critical $\dot{V}O_2$ (ml.kg^{-1}.min^{-1})**
Elite endurance-trained humans	Jones et al. [157]	12.1 ± 2.6	-	65.78 ± 2.13^{1}
Trained young humans	Barstow and Mole [158]	23 ± 12	-	-
	Berger and Jones [159]	20 ± 6	-	-
	Cleziou et al. [160]	17 ± 4	-	-
	Koppo and Bouckaert [161]	19 ± 7	-	-
	Koppo et al. [112]	15 ± 2	-	-
	Heubert et al. [162]	17 ± 9^{2}	-	-
	Jenkins and Quigley [163]	-	314 ± 28	44.8^{3} ± 4.3
	Smith et al. [164]	-	299 ± 61	39.9^{4} ± 7.9

Mean<br>SD		19<br>3	307<br>11	
Active young humans	Ferguson et al. [165]	28 ± 5	212 ± 36	24.5^{3} ± 4.2
	Murgatroyd et al. [14]	27 ± 5	236 ± 32	28.4^{3} ± 3.9
Mean<br>SD		28<br>5	224<br>34	26.5<br>4.1
Healthy elderly humans	DeLorey et al. [166]	49 ± 8	-	-
	Neder et al. [35]	-	104 ± 24	19.9 ± 2.9^{4}
	Neder et al. [34]	-	110 ± 27	21.4 ± 3.5^{4}
	Overend et al. [32]	-	115 ± 9	19.3 ± 1.2^{5}
Mean<br>SD		49<br>8	110<br>20	20.2<br>2.5
COPD patients	Nery et al. [167])	56 ± 5	-	-
	Puente-Maestu et al. [168]	85 ± 13	-	-
	Somfay et al. [169]	70 ± 8	-	-
	Puente-Maestu et al. [38]	84 ± 18	-	-
	Malaguti et al. [40]	-	41 ± 16	13.8 ± 2.1^{4}
	Neder et al. [34]	-	65 ± 14	14.7 ± 2.2^{4}
Mean<br>SD		74<br>11	53<br>15	14.3<br>2.2

Horse	Langsetmo et al. [170]	10 ± 1	-	-
	Lauderdale and Hinchcliff [24]	-	2490 ± 150	117.0 ± 7.0[6]
Mean		10	2490	117
SD		1	150	7
¤ Rat	Copp et al. [25]	-	49 ± 1[6]	68.8[8]
	Behnke et al. [171]	23 ± 1	-	-
Mean		23	-	-
SD		1	-	-
Ghost crab	Full and Herreid [23]	-	0.34 ± ???[9,10]	16.7 ± ???[9]
	Poole et al. [172]	144 ± ???[11]	-	-
Lungless salamander	Full [22]	-	0.16 ± ??? [9,10]	7.5 ± ???
	Poole et al. [172]	180 ± ???[11]	-	-

[1] Critical speed was not reported in this study, however, the speed at the lactate turnpoint and its associated metabolic rate were taken as an approximation of the critical $\dot{V}O_2$ value in these subjects.

[2] For the Heubert et al. [162] study, $\tau_{\dot{V}O2}$ was reported at four ostensibly severe work rates. Therefore the reported $\tau_{\dot{V}O2}$ value for this study is the pooled mean of the $\tau_{\dot{V}O2}$ for each work rate.

[3] To calculate a critical $\dot{V}O_2$ value, critical power in W was first multiplied by the approximate O_2 cost of cycling (i.e. 10 ml.min^{-1}.W^{-1}). This value was then divided by the reported body mass in each of these studies to provide a value free from the influence of greater body mass. Whilst it is acknowledged that the O_2 cost of cycling increases above the gas exchange threshold, it was reasoned that this method would provide a reasonable approximation of critical $\dot{V}O_2$ given the wide spectrum of aerobic function reported in table S1.

[4] In each of these studies, the metabolic rate associated with critical power was reported. Thus, the metabolic rate associated with critical power was divided by the reported body mass in each study, allowing a relative critical $\dot{V}O_2$ value to be presented.

[5] In this study, critical power was presented as a percentage of $\dot{V}O_2$ max. This enabled calculation of the metabolic rate associated with critical power, which was subsequently divided by the reported body mass to allow a relative critical $\dot{V}O_2$ value to be presented.

[6] In this study, the relative percentage of $\dot{V}O_2$ max at which the longest and shortest prediction trials were conducted was reported, along with the duration of these trials. The relative percentage of $\dot{V}O_2$ max at which critical power occurred was then calculated using the work-time model. This represented 100% of $\dot{V}O_2$ max, which was reported by Lauderdale & Hinchcliff [24] to equal 117 ± 18 ml.kg^{-1}.min^{-1}. However, it is conceded that a critical power occurring at 100% of $\dot{V}O_2$ max is highly unlikely. The longest prediction trial in this study was ~180 seconds, thus it is likely that critical power has been over-estimated in this study. However, even if critical power occurred at 80% $\dot{V}O_2$ max in this species, the hyperbolic nature of the curve is still retained. Thus, for consistency with published work, the actual values are reported.

[7] Values are for critical speed, i.e. with units of m.min^{-1}.

[8] Critical speed reported using the linear distance-time model (i.e. the model with the best fit reported in this study) occurred at 78.9% of the speed associated with $\dot{V}O_2$ max. This enabled calculation of the relative critical $\dot{V}O_2$ for this species. It is also acknowledged that calculation of $\tau_{\dot{V}O2}$ in this species might be complicated by the use of an isolated muscle preparation by Behnke et al. [171], as well as the influence that electrical stimulation has on muscle recruitment.

[9] Although in each of these studies critical power was not formally measured, time to exhaustion tests were conducted across the full spectrum of available speeds along with simultaneous measurements of $\dot{V}O_2$. Both of these papers utilised a speed above which a steady-state was attainable with time to exhaustion typically < 20 minutes. Figures of the linear relationship between $\dot{V}O_2$ and speed were presented in both papers, which enabled calculation of the $\dot{V}O_2$ associated with the "critical speed" of each species. Values for critical $\dot{V}O_2$ were then converted from $ml.g^{-1}.h^{-1}$ to $ml.kg^{-1}.min^{-1}$ to provide a value for relative critical $\dot{V}O_2$.

[10] Values are of critical speed, with units of $km.h^{-1}$.

[11] Values were taken from the 2005 review by Poole et al. [172], and thus SD's are not available. There was some discrepancy between the $\tau_{\dot{V}O2}$ values reported by Poole et al. [172] for each species and the studies that were cited in this review. The values from the review were therefore cited as these were deemed to be more reliable than any individual report.
